# Vaccine hesitancy among mobile pastoralists in Chad: a qualitative study

**DOI:** 10.1186/s12939-018-0873-2

**Published:** 2018-11-14

**Authors:** Mahamat Fayiz Abakar, Djimet Seli, Filippo Lechthaler, Esther Schelling, Nhan Tran, Jakob Zinsstag, Daniel Cobos Muñoz

**Affiliations:** 1 Institut de Recherche en Elevage pour le Développement, PO Box 433, N’Djamena, Chad; 2 Swiss Tropical and Public Health Institute, PO Box CH-4002, Basel, Switzerland; 3 University of Basel, PO Box CH, 4001 Basel, Switzerland; 4 Centre de Recherches en Anthropologie et Sciences Humaines (CRASH), PO Box 6542, N’Djamena, Chad; 5 School of Agricultural, Forest and Food Sciences, Bern University of Applied Sciences, PO Box 3052, Zollikofen, Switzerland; 6 The Alliance for Health Policy and Systems Research (AHPSR), World Health Organization, PO Box 1211, Geneva, Switzerland

**Keywords:** Immunization, Vaccination, Barriers, Mobile pastoralists, Chad

## Abstract

**Background:**

Demand side barriers to vaccination among rural and hard-to-reach populations in Chad are not yet well understood. Although innovative approaches such as linking human and animal vaccination increase vaccination uptake among mobile pastoralist communities, vaccination coverage in these communities is still lower than for rural settled populations. We hypothesize that mobile pastoralists’ communities in Chad face specific demand side barriers to access vaccination services. Understanding the factors that caregivers in these communities consider, explicitly or implicitly, in order to decide whether or not to vaccinate a child, in addition to understanding the provider’s perspectives, are essential elements to tailor vaccination programmes towards increasing vaccination acceptance and uptake.

**Methods:**

We conducted a qualitative study in a rural health district in southern Chad in April 2016 with 12 key informant in-depth interviews and four focus group discussions (FGDs) including 35 male and female participants. Participants in the study included caregivers, traditional chiefs, local and religious leaders from mobile pastoralist communities, and health officials and staff. We conducted a content analysis using a pre-defined set of categories for vaccine hesitancy covering issues on harmful effects of vaccination, mistrust with vaccination programmes/services, issues with the health system and other issues.

**Results:**

The groups of demand side barriers reported most frequently in focus group discussions were mistrust on the expanded programme on immunization (EPI) and polio vaccination outreach services (53%, *n* = 94), followed by health system issues (34%, n = 94), and concerns related to potential harm of vaccines (13%, n = 94). Concerns identified by caregivers, health professionals and community leaders followed a similar pattern with issues on programme mistrust being most frequently reported and issues with harm least frequently reported. None of the health professionals reported concerns about vaccinations being potentially harmful.

**Conclusion:**

Mobile pastoralist communities face specific demand side barriers to vaccination. Understanding these barriers is essential to reduce vaccine hesitancy and increase vaccination uptake. Local health systems must plan for the periodic presence of pastoralist communities in their zones of responsibility and create more mutual trust.

## Background

Vaccination is one of the most successful and cost-effective interventions in public health [21]. In 2015 the World Health Organization estimated that 2–3 million deaths from vaccine preventable diseases (VPD) were averted annually due to vaccination. However, vaccination provision and uptake remains low across diverse settings with substantial gradients across population groups [11, 22]. Globally, the international community has made important strides towards improved vaccination coverage [31], but in most low and middle income countries, full immunization coverage of children and women is distributed unevenly according to socio-economic status [22].

Although increasing access to vaccination has been the main strategy employed to improve vaccination coverage during the last three decades, policy makers and international institutions have more recently focused on “vaccine hesitancy” (demand side barriers to vaccination) [15]. Vaccine hesitancy exists “when vaccine acceptance in a specific setting is lower than what would be expected, given the availability of vaccine services” [23]. Vaccine hesitancy could lead to suboptimal compliance with vaccination schedules in children, low vaccine uptake or even vaccine refusal [3, 7]. A recent systematic review of the literature found that despite concerns spread across most of the settings, e.g. the fear that vaccines could produce serious negative effects on children’s health, these were highly influenced by cultural, religious or social beliefs [5].

In Chad, a sub-Saharan African country with about 14 million inhabitants [13], polio vaccination coverage (3 doses) was 32% and measles vaccination coverage was 36%, according to data from the last Multiple Indicator Cluster Survey (MICS) conducted in 2010. These figures were even lower for rural areas. Among mobile pastoralist communities, vaccination coverage among livestock was significantly higher than for children [26, 32]. Mobile pastoralists in Chad represent a particular case of inequity in access to vaccination services. A recent random household survey in two rural districts showed that health service utilization rates were relatively low on average and were systematically lower for mobile pastoralists as compared to the rural settled populations [16]. With regard to immunization, only 7% of children in pastoralist communities were vaccinated against Bacillus Calmette–Guérin (BCG) vaccine compared to 79% of children among the rural settled population. Poliomyelitis vaccination coverage was 11.6% among mobile pastoralist communities and 80% among settled children [28].

The Sustainable Development Goals (SDGs) target reduction of inequities in an interdisciplinary manner with universal policies considering the needs of disadvantaged and marginalized populations [8]. Although innovative approaches such as linking human and animal vaccination by utilizing interdisciplinary teams increases vaccination uptake among mobile pastoralist communities [2, 14, 18, 25], vaccination coverage among these groups is still lower than for rural settled populations.

Demand side barriers to vaccination among rural and hard-to-reach populations in Chad are not yet well understood. Based on anecdotal and non-structured analysis of people’s beliefs and attitudes towards vaccination, managers of the Expanded Programme on Immunization (EPI) focused their efforts on providing information on the benefits of vaccines as a means to increase vaccination uptake. However, evidence from other West and Central African countries suggests that there can be other relevant factors that prevent caregivers from vaccinating their children, for instance, concerns about the potential harm of vaccines in Benin [9], fear of vaccination in Nigeria [1] or worry about health staff being unpleasant in Burkina Faso [27]. Lack of transportation, language barriers and health staff being unpleasant are major constraints preventing pastoralist women from accessing health services in Chad [10]. For Muslim women, the necessity to obtain a husband’s permission is a further important challenge [6], as men are often absent due to work obligations.

This study uses a qualitative approach to identify demand side barriers which hinder access to vaccination services among mobile pastoralist communities in Chad. Qualitative methods allow exploring the underlying issues underlying issues of vaccination hesitancy beyond the observed low utilization rates of vaccination services among mobile pastoralist communities. We hypothesized that Chadian mobile pastoralist communities face specific demand side barriers to access vaccination services. Understanding factors which caregivers in these communities might consider, explicitly or implicitly, in order to decide whether or not to vaccinate a child is an essential element to tailor vaccination programmes to increase vaccination acceptance and uptake. To our knowledge, the present study is the first to investigate factors that prevent mobile pastoralists from accessing vaccination services in Chad, providing an important opportunity to advance the understanding of why caregivers in hard to reach communities might decide not to vaccinate their children. The findings of this study will also inform the design and implementation of interventions to increase vaccination outcomes among mobile pastoralist communities. In this paper, the term “nomadic communities” is used interchangeably when referring to the same communities despite the fact that not all mobile pastoralists are necessarily nomads.

## Methods

### Data collection & participants

Data was obtained from 12 individual semi-structured interviews and four focus group discussions (FGDs).

Selection of participants was based on a convenience sampling approach designed to maximize demographic and functional diversity of the sample around the topic of vaccination. Interview participants were recruited based on their availability and included primary caregivers (mothers with at least one child under 5 years old), heads of families who were decision makers and resource controllers, local health authorities and civil and religious leaders (Table 1). Participants in the FGDs were mothers and heads of the family (Table 1). We combined the perceptions from the different groups for the analysis.

**Table 1:** Summary of data collection

	Interviewees	Sex	Location	Number
Individual interviews	Civil authority	Male	Danamadji	1
	Responsible for vaccination services	Male	Danamadji	1
	Head of family	Male	Ridina, Konoko, Daranaïm, Djanatanaïm Ridina, Konoko	5
	Mother with child < 5 years	Female	Daranaïm	4
	Religious leader	Male		1
Total interviews				12
FGD	Mothers with child < 5 years	Female	Ridina, Daranaïm	2
	Head of family	Male	Darbarid, Alqaba	2
Total FGD				4

First drafts of the interview and FGD guidelines were based on literature review and discussions with health experts in the Chadian context. The draft was field tested and further refined in a participatory process involving local health experts and representatives from the nomadic communities to compare and contrast views and opinions. Each FGD took approximately one hour and was facilitated by at least two people of the same sex as the participants. Individual interviews took between 40 and 60 minutes. Data from male participants was obtained in mobile pastoralists’ camps, in a quit and private place away from distractions. To ensure confidentiality and a favorable atmosphere for discussions, interviews and FGDs with nomadic women were conducted in a protected environment, most often in a woman’s home. Data collectors were trained anthropologists and sociologists from the University of N’Djaména holding at least a Master’s degree. No relationship existed between researchers and study participants prior to study commencement.

### Analysis

Face-to-face interviews and FGDs were audio recorded, transcribed and translated from Chadian Arabic into French. Data evaluation was done manually following a deductive approach. More specifically, the analysis of the transcripts was based on content analysis using a directed approach [12, 29] based on pre-defined categories of vaccine hesitancy. A pre-defined framework to understand concerns about vaccinations [5, 17, 24] was adapted to first code and then categorize the themes. The categories were: (i) issues with harmful effects of vaccination, (ii) issues with programme mistrust, where programme was defined as all vaccination activities carried out (e.g. Expanded Programme on Immunization “EPI” and polio vaccination outreach activities), (iii) health system issues, and (iv) other issues raised as shown in Table 2, specific sub-categories were used to further refine the categorization.

**Table 2:** Categories of concerns about vaccination adapted from [5, 17]

1. Issues with harmful effects of vaccination	
a. Concerns that immuno-compromising	
b. Concerns that causes diseases / general harm / adverse effects	
c. Concerns that is harmful if the child is sick	
d. Concerns with side-effects of vaccination (including pain)	
e. Parents remember their own or other adverse experiences	
f. Exposed to pathogens in clinics	
g. Fear of needles	
h. Vaccines are provided at too young age / too many vaccines and doses	
2. Issues of mistrust with vaccination programmes/services	
a. Rumours / mistrust of medical community	
b. Lack of confidence in vaccines effectiveness	
c. Religious reasons	
d. Not enough information to make the decision	
3. Health system issues	
a. Concern with costs / access to the health facility	
b. Concern with time / working hours of services	
c. Health staff are unpleasant / untrained	
d. Concern with not being able to get vaccination	
e. Concern with quality of the vaccines	
4. Other issues	

First, transcripts were carefully reviewed to highlight all text that appeared to describe vaccination concerns. Then, all highlighted text was coded using the pre-defined categories. Text that did not fit into one of the categories was assigned as “other issues”. Study findings were reported based on the incidence of codes that represent the pre-defined categories by comparing the overall rank order of the codes and the corresponding percentages within and between the population groups [5]. Evidence was further described using selected verbatim quotations from research participants. Due to high illiteracy rates in rural Chad, transcripts were not returned to the study participants.

### Ethical considerations

Ethical clearance was given by the National Bioethics Committee in Chad “Comité National de Bioéthique du Tchad (CNBT)” and the WHO Research Ethics Committee Review (WHO ERC) (Décision N°186/PR/PM/MESRS/SG/CNBT/2016, 12/04/2016; Protocol ID: ERC.0002684, 18/03/2016).

The study participants were given detailed information about the purpose of the study, the background of the data collectors and the extent of their involvement. Informed consent was obtained from all study participants before starting any interviews or FGDs. It was explained that participation was voluntary, without compensation, individuals could withdraw from the study at any time, statements would be anonymised so that attribution to individuals would not be possible, and participation was without negative consequences for them, their family or their community. All participants were adults and either signed or fingerprinted the consent form.

## Results

A total of 94 coded quotes that reported concerns about hesitancy or barriers to access vaccination in Danamadji were extracted from the transcripts based on 12 individual interviews and four FGDs. In total, 35 men and women participated in the four FGDs. There were no refusals to participate in the study.

The quotes were retrieved as follows: 73 from interviews, 15 from FGDs with male participants and 12 from FGDs with female participants. Most quotes were from caregivers, followed by health staff and community leaders. We present the results using the pre-defined categories to describe the results [5, 17] issues with harmful effects of vaccination, issues with mistrust with vaccination programmes, and issues with the health system.

### Demand side barriers to vaccination in Danamadji

**Fig. 1:**
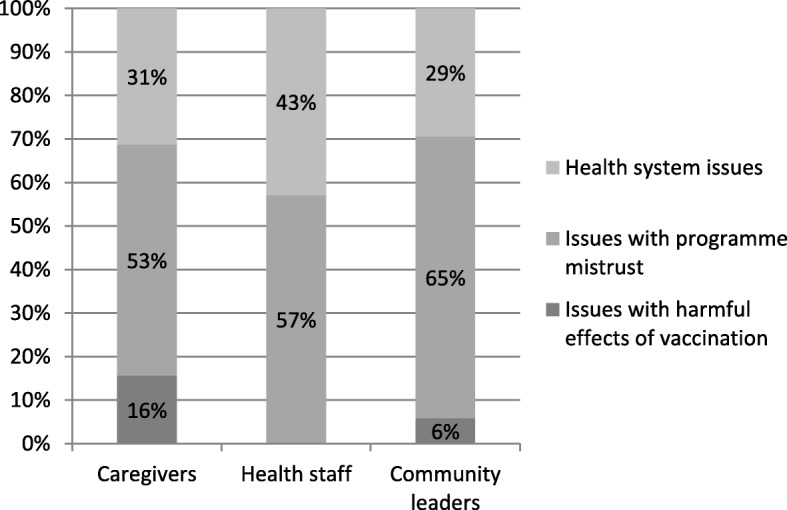
Distribution of concerns about vaccination reported in Danamadji, according to participant type

The most frequently reported demand side barriers were related to issues with programme mistrust (53%, *n* = 94), followed by health system issues (34%, *n* = 94), and concerns related to potential harm of vaccines (13%, *n* = 94). The sub-categories with the five most frequently demand side barriers to vaccination are shown in Table 3. “Not enough information to make decision” is the most frequent concern followed by “Health staff being unpleasant/untrained”, “Religious reasons”, “Concerns that causes diseases/general harm/adverse effects”, and “Concerns with time/working hours” (Fig. 1). None of the health professionals reported concerns about vaccination being harmful.

**Table 3:** Five most frequently reported demand side barriers to vaccination in Danamadji district

Category	Issue/concern	Number of quotes
Trust/mistrust	Not enough information to make the decision	34
Health system	Health staff are unpleasant / untrained	22
Trust/mistrust	Religious reasons	10
Harm	Concerns that causes diseases / general harm / adverse ffects	9
Health system	Concern with time / working hours	6

### Issues with harmful effects of vaccination

Concerns related to the potential harmful effects of vaccines or their side effects were the least reported category of demand side barriers to vaccination among mobile pastoralist communities in Danamadji. Among other concerns, participants reported the risk of sterility, fever, diarrhoea, inflammation of the throat and even death.

For some individuals, the prevention of certain diseases by vaccines was not a desired outcome because diseases were opportunities for the body to get stronger and, since vaccines prevented them, they subsequently weakened the child’s (immune) system. One father pointed out:

“*In general, diseases are part of human daily life. It is not all the time that when a child is sick, then we should go immediately to a hospital. Diseases for children are somehow a necessary bad thing. By falling ill, they become immune. So we do not go to the hospital or health centre immediately for a given disease of the child*” (Man, Alqaba).

Some extreme views were reported by caregivers who believed that one of the reasons why children die unexpectedly was because they were consistently healthy:

“*Certainly the disease is a very bad thing, especially malaria and colds in children. In general, we do not want the children to get sick. (…) But when the child is still in good health, there is fear the day it falls ill. I have in all 7 children, 2 of them died. One died unexpectedly because he was always healthy. One day he fell ill and the third day of his illness, he could not conquer the disease and died. Therefore, diseases in children are a sign of longevity. Do not regret too much that children fall ill*” (Woman, Konoko).

### Issues of mistrust with vaccination programmes/services

The most frequently reported demand side barrier to vaccination were that caregivers did not have enough information to understand the benefits of vaccination and to know the process for getting their children vaccinated or they did not trust health workers coming to the camps during polio outreach vaccination days. It was widely acknowledged by most participants that nomadic communities received too little information about vaccines and their benefits:

“*We cannot comment on the pros and cons of a thing until you know the thing. In the case of vaccination, we cannot say, because no one came to us to tell us against which disease the vaccination is. So we cannot know whether vaccination is a solution as described by the State or a problem as the rumours say*” (Male, Alqaba).

There was some confusion about the difference between vaccination days for polio vaccination and routine vaccination. Several participants referred only to polio vaccination when raising their arguments. They usually talked about “drops” when they referred to vaccination in general. Benefits of vaccination were not clear, and there was some reluctance to believe that vaccines are effective:

“*Yes we heard about the vaccination about polio. But also for this vaccination issue, we have not seen drugs that can cure or protect our children*” (Female, Konoko).

Even the most basic information about vaccinations did not reach some nomadic communities:

“*Vaccination, I heard about it in street talks from people who do not know more than me. So nobody really told me what it is”* (Male, Darannaïm).

No communication channel was reported that brings pro-vaccination messages to nomadic communities. Some caregivers complained that instead of receiving more information they were intimidated by health authorities forcing them to vaccinate their children. A camp leader reported:

*“There are no communication channels between nomads and health professionals. In our camp here as elsewhere, we do not have a person who acts as an intermediary to convey information between us and the immunization services. There is a total lack of dialogue between us and the health services. They do not even have the phone number of the camp chief to call and deliver information”* (Male).

Mobile pastoralist communities were usually reached less often with information than other communities, and they did not have regular contact with health facilities or authorities. There were only few opportunities when individuals had regular contact with conventional health services (e.g. antenatal care visits or delivery), and they only made use of health services during illness or in emergencies. A participant in a FGD was surprised that health services could be used preventively:

“*Here we do not know that when a child is healthy, we can get him to the hospital to take the vaccine which protects him against a number of diseases that are disturbing and even killing our children*” (Male, Darbarid).

Even when health workers from the district health system moved to camps to provide health services, they did not use the opportunity to reduce the information gap with either local leaders or caregivers. A mother complained:

* “What we deplore is that when the vaccinators come here, they do not explain what they came to do. They only call the children and put the drops in their mouths. They are always in a hurry.”* (Female, Daranaïm).

Some participants pointed out that it is understandable that parents do not have trust in something they do not know or have not been informed about. The way in which polio outreach vaccination days are organised, with teams moving from camp to camp during one day to vaccinate children, was inadequate to provide sufficient information and convince caregivers to vaccinate their children, specifically for routine immunization. A religious leader reported:

“*It’s true that I am the imam* (religious leader) *having a great influence on the community, but no one came to explain to me what the vaccination is about so that I may have the possibility to widely inform my congregation during the Friday prayer and when people meet. General health services and those related to vaccination in particular lack a method to inform or convey their message. For example in the case of polio, instead of explaining us the merits and inform us of the arrival of the team of vaccinators in our camps, we suddenly see them arriving and catching children within their reach and administering the drops of vaccines. We parents only accept this method against our will. Some prefer to hide their children and sometimes colour their fingers to show that the children were already vaccinated so that they would not swallow anything. All of this is the lack of information about polio*” (Religious leader).

Religious and cultural beliefs also played a role in the decision making process of whether to vaccinate infants living in nomadic camps. Some communities believed that diseases are “(…) *a plan of God to train the child for the difficult moments*” (Woman, Daranaïm), and they should not fight them. For others, diseases had a religious/divine origin, and they must be handled by traditional healers such as the Marabout. The services provided by traditional healers were considered to be more affordable, despite the fact that vaccination is offered free of charge in Chad. They were also considered more effective as compared with western medicine (including vaccination):

“*When the children here become sick, I intervene in the first place to use religious incantations. And it is only when the disease persists that the child is brought to the hospital. There are also cases where the child is sent home from the hospital without being cured, and at this level too I am the last resort to pray for God to cure the sick child. I intervene, I use incantations for all kinds of diseases*” (Male [Marabout], Ridina).

### Issues with the health system

Concerns about vaccination providers in health facilities being unpleasant were widely reported by participants in interviews and FGDs. Some participants considered health workers to be authoritarian and bad tempered. One informant pointed out:

“*When we go to the health centre to seek care for our sick children, we are very poorly received. In some situations, we are expelled by health workers. Hoping that a child would never be ill so that one would not have to wait at the hospital saying that we want to have medication, imagine what treatments can be reserved for us? We pastoralists, don’t we have rights? Health workers consider us like our animals”* (Male, Konoko).

Nomadic communities had a very poor opinion of health care workers in the district which was a major barrier to vaccination. Even acknowledging that vaccination is free of charge and could have some benefits for children, parents decided not to vaccinate because they did not trust the system that provides them:

“*You see filthy agents who come to do this work. Some of them are drunkards, which we know. This does not inspire confidence. You end up saying this vaccination business is a way to get work for some, to make money for others and all this at the expense of the health of our children. Finally, I have a negative perception of the entire vaccination operation*”, (Male, Ridina).

In line with this idea, *s*ome participants believed that vaccination was not effective and vaccination campaigns were only conducted in the interest of district officials and health workers who receive per diem payments for the vaccination days:

“*For me, polio is an organised business from the high hierarchy to the last vaccinators who administers the drops. Everyone finds his interest and that’s it. This practice makes me doubt about the efficacy of poliomyelitis vaccine*” (Male, Darannaïm).

It was frequently reported that health professionals working in vaccination campaigns were not trained doctors or nurses, which raised concerns about their capacity to deliver vaccination activities. Some interviewees even saw this situation as a form of disrespect to nomadic communities:

“*They send us the dirty young people from the neighbourhood to come and vaccinate our children against poliomyelitis. They are young people who only seek money without further concerns. They do not know anything about health (…..) People treat us like our animals and have no consideration for us. We have never seen a trained health worker or a doctor coming to vaccinate our children”* (Man, Ridina).

The fact that the messages about vaccination (essentially polio) or the interventions themselves were not provided by staff who were considered as peers in terms of religious and cultural background prevented some parents from vaccinating their children. In addition to the language barrier between health professionals and nomadic communities, health workers seemed to ignore the basic cultural and religious norms in the camps, and there were few Muslim health workers. One camp chief criticised:

“*We want people to send us staff who know the health field. We prefer people close to our realities, who know our problems and who can transmit them to higher authorities to react. We want reliable people, who can educate us on vaccination*” (Camp leader, Male)*.*

Other examples of health system issues were the lack of cultural appropriateness of vaccination services and the timing of teams from the health centre for visiting the camps. The timing for the visits significantly interfered with daily activities and obligations for both men and women in the camps. As one of the administrative authorities in Danamadji recognised,

“*The reality is that the vaccinators cannot make the trip to the camps at any time, when the head of household is absent following his herds, for example. This is a serious cultural breach in pastoral communities as a wife has to seek permission of her husband to decide on certain issues; such as exposing a child to strangers. This is also valid for the vaccination of children*” (Male, Danamadji).

## Discussion

We identified demand side barriers to vaccination among nomadic communities in Danamadji District in Chad. We conducted a study using qualitative research to identify factors or beliefs that prevent caregivers from vaccinating their children. Contrary to expectations, this study found that issues related to the health system itself, such as health staff being unpleasant or vaccination campaigns being poorly organised, were widely reported by nomadic communities. In comparison to previous studies on vaccine hesitancy in low and middle income countries (LMIC) [5], it is surprising that nomadic communities did not raise many concerns about vaccines being harmful for children or causing dangerous adverse effects. A recent literature review synthesised concerns about vaccination in LMICs and found issues with harmfulness were the most frequently reported in both qualitative and quantitative studies across all continents [5]. In the current study, however, the experiences reported on vaccination of children were virtually exclusively for polio vaccination. Other vaccines such as the pentavalent vaccine are more prone to cause side effects than the polio vaccine. Another interesting finding is that the Muslim nomadic communities did not report concerns about vaccines being part of a (Western) conspiracy to harm or sterilise Muslim communities. These concerns have been widely reported in Muslim countries [4] as a major barrier to vaccination [20].

There is a significant body of literature on barriers to vaccination services which shows that concerns about the harmful effects of vaccination and lack of information are the most important (frequently reported) demand side barriers to vaccination [7, 15, 17]. However, our study shows that nomadic communities face specific barriers to vaccination that must be known in order to adapt the provision of services at the local level. This study reveals specific barriers for mobile pastoralist communities, particularly mistrust of and bad reception at health centres that were not captured in previous studies. This mismatch might be explained by the fact that a substantial number of studies used structured questionnaires to quantify the proportion of respondents with a specific concern or its impact on vaccination behaviour [5]. Many of the questions from these questionnaires focused on these demand side barriers (harmful effects and vaccine knowledge among users) rather than exploring concerns more widely. As a result of the approach there is an artificial overestimation of them based on increased reporting. Hence, our study supports the use of qualitative research to improve the design of behaviour change communication campaigns.

A limitation is that most participants referred primarily to the polio vaccination outreach days in their answers although the questions were designed to capture the perception of routine and outreach vaccination services. Issues related to lack of trust in the vaccination outreach days against polio and health system issues were the most frequently reported. Nomadic communities were reluctant to vaccinate their children because they did not have enough information about the benefit of vaccines or the location of vaccination services in their district. A substantial number of participants in our study complained that health staff were unpleasant or that they felt neglected or humiliated because they were nomads. They raised concerns about the qualification of vaccinators and their cultural distance from the nomadic community as one of the most important disincentives to get their children vaccinated. They sought interlocutors with knowledge on the daily life and culture between them and the health services.

Communication channels between nomadic communities and the local health system does not seem to exist. It is well known that communication and social mobilisation is associated with high vaccination coverage and allows for reaching “sparsely covered groups” when included as a key component of immunisation programmes, e.g. polio [19, 30]. Although standard communication campaigns exist, participants in our study stated that there was essentially no exchange of information taking place between mobile pastoralist communities and the local health system. In addition, the few existing information channels were not adapted to the pastoralist context, where illiteracy rates were much higher than among settled communities, and did not address the real concerns of nomadic communities’ caregivers in the Danamadji health district. Therefore, advocacy could be directed towards exploring new channels of communication with nomadic communities in the Danamadji health district by involving the community more through local social mobilisation teams and maintenance of a permanent contact network, e.g. using mobile phones with camp and group leaders. Importantly, interlocutors between them and the services need to be established.

The health system in Danamadji fails to provide vaccination services to nomadic communities, which translates into substantial immunisation inequities when compared to rural settled populations in the same district. To reduce health inequities in light of the SDGs, modern health system interventions must ensure that all population strata benefit from new policies. Barriers to existing vaccination services among mobile communities in Danamadji could be substantially reduced by improving the information exchanges between the provider (health district) and the population. It has been previously shown that outreach campaigns combining veterinary and human health services are a promising and feasible strategy for increasing vaccination uptake among mobile communities [25]. These campaigns were also successful because there was and is on-going investment in appropriate information campaigns based on perceptions of human and animal vaccination. Adapted information material and routine EPI vaccination campaigns using interlocutors for dissemination are on-going in Danamadji.

In the past, we have made good experience with trans-disciplinary stakeholder meetings [26] between communities, authorities and health workers. Such meetings are foreseen in this study to feedback the results and plan for Information, Education and Communication (IEC) training sessions focusing on building empathy and cultural sensitivity among the participating health workers.

A limitation of this study is that during transcription interviews were translated from Chadian Arabic to French. Even though the translation was done by native Chadian Arabic speakers, there was still potential for lost meaning during the process.

## Conclusions

Nomadic pastoralist communities face specific demand side barriers to vaccination. Understanding these barriers is essential to reduce vaccine hesitancy and increase vaccination uptake. Local health systems must be sensitive to and act upon these specific needs, particularly the mistrust based on bad experiences of mobile pastoralist communities.

## Abbreviations

AHPSR: The Alliance for Health Policy and Systems Research
*BCG*: Bacillus Calmette–Guérin *vaccine*
CNBT: Comité National de Bioéthique du Tchad
CRASH: Centre de Recherche en Anthropologie et Sciences Humaines
EPI: Expanded Programme on Immunization
FGDs: Focus Group Discussions
IEC: Information, Education and Communication
INSEED: Institut National de la Statistique, des Études Économiques et Démographiques
IRED: Institut de Recherche en Elevage pour le Développement
KII: Key informant in-depth interviews
LMIC: Low and Middle Income Countries
MICS: Multiple Indicator Cluster Survey
PADS: Projet d’Appui aux Districts Sanitaires au Tchad: Yao et Danamadji
RCA: Republic of Central Africa
SAGE: Strategic Advisory Group of Experts on Immunization
SDGs: The Sustainable Development Goals
Swiss TPH: Swiss Tropical and Public Health Institute
TD: Transdisciplinary
VPD: Vaccine preventable diseases
WHO ERC: WHO Research Ethics Committee Review
WHO: World Health Organization
